# Dental education evaluation in China: a systematic review

**DOI:** 10.1186/1472-6920-14-178

**Published:** 2014-08-27

**Authors:** Jingyi Yang, Ying Zhang, Xiaolong Ye, Cheng Xie, Xv Ge, Fan Lu, Qing Yu, Hantang Sun

**Affiliations:** Division of Training, the Fourth Military Medical University (FMMU), Xi’an, China; State Key Laboratory of Military Stomatology, Department of Operative Dentistry and Endodontics, School of Stomatology, the Fourth Military Medical University, 145 West Chang-le Road, Xi’an, 710032 China; State Key Laboratory of Military Stomatology, Division of Medical and Training Affairs, School of Stomatology, FMMU, Xi’an, China; Department of Biochemistry and Molecular Biology, FMMU, Xi’an, China

**Keywords:** Dental education evaluation in China (DEEC), Accreditation on dental education in USA, Evaluation standards, Evaluation actions, Site visit

## Abstract

**Background:**

Accreditation of education is very important for maintaining and improving education quality. With the development of modern dental education, more and more attention is being paid to accreditation of dental education in China. Current accreditation of dental education in China is called “dental education evaluation”. By using a systematic review, this paper aims to provide the general profile of the standards and process of dental education evaluation in China (DEEC).

**Methods:**

A systematic review on DEEC was performed, CAJD and VIP databases were employed to identify all literatures which were relevant to DEEC. Profile and features of DEEC were compared with those of the Accreditation Standards for Dental Education Programs of the USA (ASDEPU).

**Results:**

The current standards for the evaluation are composed of six modules and twenty-four items, the evaluation process consists of three stages There was some difference between DEEC and its American counterparts.

**Conclusions:**

Accreditation on dental education is very important for the maintenance and improvement of education quality. As the primary form of dental education accreditation, DEEC is basically suitable for current dental education conditions in China, however, in order to keep pace with the changing conditions, both the standards and actions of DEEC should often be revised.

## Background

Dental education in China has a history of more than 1400 years. In the early stage of the Tang Dynasty, a four-year program in specialty of dentistry was opened by the Imperial Medical Academy, which was regarded as the earliest formal institution for Chinese traditional dental education [1]. Modern dental education was introduced into China in 1917, when the first dental school, the Faculty of Dentistry, West China Xie He University, was established in Chengdu, Sichuan Province. Since the 1980s, with China opening up and developing rapidly, more academic dental institutions have been established, and more dentists have been produced, so that the increasing needs for oral health care of the public could be meet [2, 3]. With the dramatic increase of the dental schools and dental undergraduates in China, the need of dental education quality assurance was proposed. In China, current high education quality assurance and accreditation system is called “education evaluation” [4], with “dental education evaluation in China (DEEC)” as part of it. In 2008, the draft of Accreditation Standards for Undergraduate Dental Education Programs in China (ASUDEPC) was put forward by Society of Dental Education, Chinese Stomatological Association [5], and the modified ASUDEPC (MASUDEPC) has been used in the first round DEEC. This paper aims to introduce briefly the current profile of DEEC to dental educators in the world.

## Methods

**Figure 1:**
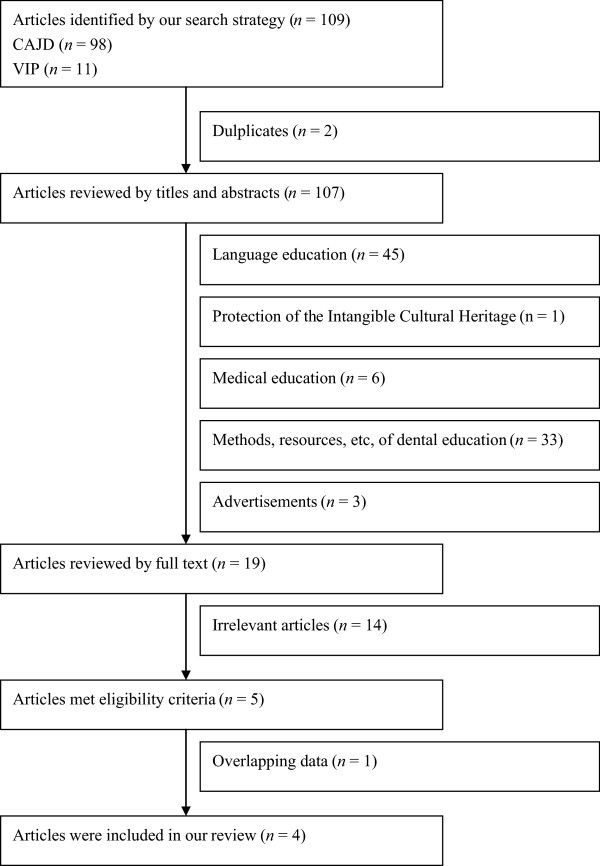
**Flowchart of study inclusion.**

A systematic review on DEEC was performed. All relevant literatures were searched via the CAJD (China Academic Journal Network Publishing Database) and VIP databases (from the database inception to July 21, 2013). Search items were listed as follows: (“stomatology” [All Fields] OR “dentistry” [All Fields] OR “oral” [All Fields]) AND (“education evaluation” [All Fields] OR “education assessment” [All Fields]). There was no publication date or publication status restriction. Literatures which were related to the standards, process, methods, preparation and results of DEEC were included, while those irrelevant to one of these aspects of DEEC were excluded (such as the studies on the evaluation of some distinct teaching methods) (Figure 1). The following data from included literatures was extracted: the first author, publication year, institution where DEEC was performed, if possible, preparation , self-assessment reports and results of the evaluation was also extracted.

## Results

### Profile of evaluation standards of DEEC

The MASUDEPC is a simplified version of ASUDEPC, to some extent, it is a combination of ASUDEPC and the current accreditation standards for other programs in general universities. The MASUDEPC is composed of six modules: institutional doctrine, faculty and staff, development of program, educational environments, administration of the education and, educational outcome, each module having several items, as shown in Table 1.

**Table 1:** **Modules and items of the MASUDEPC**

Modules	Items
1. Institutional doctrine	
	1.1 Education idea
	1.2 Education reformation
2. Faculty and staff	
	2.1 Team structure
	2.2 Achievements
	2.3 Teaching activities
	2.4 Faculty development
3.Development of program	
	3.1 Program design
	3.2 Curriculum design
	3.3 Practice design
4. Educational environments	
	4.1 Facilities and equipments
	4.2 Information environments
	4.3 Academic environments
	4.4 Cultural environments
	4.5 Financial support
5. Administration of the education	
	5.1 Attention from the institution leaders
	5.2 Administrational organization
	5.3 Administration environments
	5.4 Administration staff
	5.5 Education quality supervision
	5.6 Education support
6. Educational outcome	
	6.1 Quality of lecture and/or laboratory work
	6.2 Students’ knowledge level
	6.3 Students’ integration capacity
	6.4 Students’ ethics and professionalism

### Module one: institutional doctrine

This module is equivalent to “standard 1: institutional effectiveness” in the Accreditation Standards for Dental Education Programs of the USA (ASDEPU) [6]. It consists of two items: education idea and education reformation. For education idea, the standards suggest that a dental institution state its education purpose clearly, provide an ongoing plan for the assessment of educational quality and improvement on program effectiveness. For education reformation, determined reformation ideas, measures to be taken and achievements acquired are asked to be provided.

### Module two: faculty and staff

Evaluation standards for the teachers are provided in this module, which are divided into four items: team structure, achievements, teaching activities and faculty ongoing development. Not only the name of the module, but also the contents of the items are almost the same as the counterpart parts in the ASDEPU. However, in the MASUDEPC for the teachers, their achievements, especially research achievements such as amount of responsible research grants, papers published, etc., are emphasized, while their participation in the school’s decision-making processes is not mentioned. Contrary to those, in the ASDEPU, teachers’ participation in the school’s decision-making processes is emphasized as a separate item.

### Module three: development of program

Both in the MASUDEPC and ASDEPU, this module is regarded as the most important one because it describes the standards set for the core part of dental education, e.g., the dental education programs.

In the MASUDEPC, there are three items in this module: program design, curriculum design and practice design. In program design, the institutions are asked to provide written information about the goals and requirements of each course, the plans to meet the goals and requirements, and the current situation of the courses. The item of curriculum design focuses on detailed affairs, such as the standards of the subjects, measures taken for the improvement of both content and textbooks of the subjects, as well as the methods of outcome evaluation. And for dental education, more attention is paid to the item of practice design, dental schools are urged to ensure not only the opportunity for the students to perform laboratory work before internship rotation, but also the availability of adequate patient experiences that enable all students to achieve their stated competencies.

### Module four: educational environments

Unlike “standard 4: educational support services” in the ASDEPU in which student financial aid and health service are emphasized, educational environments in the MASUDEPC includes a diverse series of points: facilities and equipments, information environments, academic environments, cultural environments, financial support.

The item of facilities and equipments deals with standards for the equipments for teaching, sports, laboratory work and internship rotation practice. Information environments include the construction and use of library, the establishment and use of local area network (LAN), the development and integration of information resources, and the establishment and use of simulation laboratory. The standards for academic environments are used to evaluate what kind of climate for teaching and learning has been formed, e.g., how enthusiastic the teachers and the students are towards dental education. And those for cultural environments aim to evaluate humanistic culture education. The reason for financial support to be listed as a separate item is probably that, unlike research and clinic work, the outcome of education affairs is so slow and so difficult to measure, that it can not add much scores to the achievements of the administration, therefore, to ensure that there is enough financial resources for dental education, the standards for financial support is clearly stated as a separate item.

### Module five: administration of the education

Compared with the ASDEPU, this module is a China-specific one, since there is neither counterpart module nor counterpart items in the ASDEPU.

There are six items in this module: attention from the institution leaders, administrational organization, administration environments, administration staff, education quality supervision, education support. In China, the administration team of the dental institution manages all kinds of affairs, including the affairs related to not only education, but also clinic and research. Because the outcome of education is slow and hard to measure, educational affairs are apt to be underestimated. To avoid this situation, basic requirements for education management are formulated in the MASUDEPC. For example, in the item of “attention from the institution leaders”, institution leaders are asked to discuss the educational affairs periodically, and to supervise some amount of lecture and/or laboratory work in each semester. Of “administration staff”, staff members who are in charge of education affairs are urged to do some education-related studies and to publish their results in the form of papers.

### Module six: educational outcome

In the ASDEPU, standards for expected outcome of dental education are included in standard 2-educational program, while in the MASUDEPC, standards for outcome are listed as a separate module.

There are four items in this module, one is for the teachers and the other three are for the students. The item “quality of lecture and/or laboratory work” sets standards for the evaluation of performance quality of a teacher with regard to his lectures or his ability to organize a laboratory work course. The other three aim to evaluate whether the students are up to the expected level of knowledge, capacity, ethics and professionalism.

### Process and methods of the evaluation

A cycle of evaluation consists of three stages: self-assessment by the institution itself, formal evaluation by superior administration, and the point-to-point improvement stage by the institution. According to Ministry of Education of China, the end of a cycle means the beginning of the subsequent cycle. So far, the first cycle of nationwide evaluation has just finished.

### Stage one: self-assessment

At this stage, a dental institution should check whether its current educational conditions can meet the evaluation standards by point-to-point check. If there is no self-confidence for one or more items, special efforts would be made to improve these items. For example, if a dental school is not satisfied with the academic level of lectures given by its faculty members, the training of the faculty members will not stop until satisfactory results have been achieved. The problem is that, as far as some parameters are concerned, the ambiguity of the description of the standards makes it impossible for anyone to tell whether current levels can meet the evaluation standards, therefore, efforts aiming at improving these parameters will not stop until the second stage comes.

### Stage two: formal evaluation by superior administration

In this stage, a site visit committee will enter the dental institution, and will use different kinds of methods to perform the evaluation. The members of the committee are chosen by the superior administration, for example, for the dental education evaluation of School of Stomatology, the Fourth Military Medical University, the committee members will be appointed by headquarters of the People’s Liberation Army; for other non-military dental schools, the committee members are generally appointed by Ministry of Education of China.

To make the evaluation better, the following methods are often used by the committee, the details of these methods are summarized in Table 2.

**Table 2:** **Details of methods used in site visit**

Methods	Details	Modules checked
Listening to report	Listen to the report about the overall profile of the institution	Module 1
On-site inspection	Review of program documentation without prior notification	Module 2, 3, 5
Listening to lectures and laboratory work courses	Listen to some lectures and/or laboratory work courses without prior notification	Module 2, 3, 6
On-the-spot investigation	Check the education support facilities, such as library, simulation laboratory, etc.	Module 4
Interview, survey, test, etc.	Randomly pick up institutional personnel for interviews, surveys and tests	Module 1-6

#### Listening to report

The dean of a dental school will present them with an overall profile of dental education in the school, and the committee members will surely get some information from the report, especially the evidences related to module 1: institutional doctrine.

#### On-site inspection

The members of the committee may go to some departments of the school to check the education-related files without prior notification. This method is often used to evaluate the levels of module 2, module 3 and module 5.

#### Listening to lectures and laboratory work courses

The members of the committee may randomly choose some lectures and/or laboratory work courses without prior notification, they listen to these courses and give their evaluation. This method is often used to evaluate the levels of module 2, module 3 and module 6.

#### On-the-spot investigation

The members of the committee may go to check whether the education support facilities, such as library, simulation laboratory, etc., are appropriate as education support. This method is often used to evaluate the level of module 4.

#### Interview, survey, test, etc

The members of the committee may pick up randomly some faculty and staff members, students and other related personnel for some kinds of interviews, surveys and tests, just to get more information from them. These methods are often used to evaluate the levels of all modules.

### Stage three: measures for the improvement of dental education

After a careful investigation, the obtained information will be summarized to form a conclusion on the current dental education of a dental institution. Both the positive and negative comments will appear in the conclusion report. The subsequent mission for the dental institution to accomplish is to take measures to overcome the weaknesses that have caused the negative comments, so that better scores can be obtained in the next cycle of dental education evaluation.

## Discussion

According to Tedesco LA, history of accreditation in dental education in the USA could be divided into four eras: era of stability (1940–70), era of flexibility (1970–85), era of specificity (1985–95) and, era of standards simplification (1998–present) [7]. After detailed comparison, it can be found that current dental education evaluation in China is approximately equivalent to “the era of flexibility” of that in the USA. Flexibility allows differences between dental institutions, so it is suitable for the current situation of dental education in China: different developmental levels of dental education in diverse areas [8, 9], and dental education transformation is being carried out by most of the dental institutions [9–12]. However, flexibility also has some adverse effects, with ambiguity as the most critical one. As mentioned, the ambiguous description of the evaluation standards confuses the dental institutions, they are not self-confident about whether their preparations are good enough for site visit by the committee, therefore, significant amounts of faculty and administration time, together with excessive costs, would be occupied by such preparation until the committee enters the campus. This problem also used to appear in accreditation of dental education in the USA [13].

To overcome the problems, the accreditation standards should often be reviewed, emended and modified to come up with the changing situation, just as its American counterpart has experienced and is experiencing [13]. For example, to avoid or decrease the financial and human resource waste in the preparation for site visit, the description of the standards should be more explicit. However, balance must be kept between explicitness and flexibility, so that a little room could be left for institutions to adapt to local situations and transformations.

Another concern is about the selection of the members of the commission of evaluation and site visit team. In the USA, membership of Commission on Dental Accreditation (CODA) reflects a wide geographic distribution and includes both genders and under-represented ethnic groups. According to American Dental Association (ADA), there are 30 members on the Board of Commissioners, members of the Commission are selected by the participating organizations, the current CODA representatives are summarized in Table 3. Not only the Board of Commissioners, but also the consultants who serve as site visit team members reflect a wide representative distribution: there is an extensive list of consultants in CODA’s accreditation activities, each year, individuals will be nominated to serve as consultants by the participating communities of interest, the Commission, based on qualifications and current needs for program type and geographic representation, will appoint consultants selected from those nominated persons [14]. While in China, so far there is no national organization responsible for DEEC, the senior administrations of the dental institutions are responsible for DEEC actions. Unlike those in the USA, there are no general public or students in the team, furthermore, most of the team members’ specialties have nothing to do with dentistry. So in the future, it is suggested that the selection of the committee members should be improved.

**Table 3:** **Summary of CODA Representatives**

Organization	Amounts of members
American Dental Association	4 members
American Association of Dental Boards	4 members
American Dental Education Association	4 members
Postdoctoral General Dentistry	1 member
Recognized Dental Specialties (one each)	9 member
American Dental Assistants Association	1 member
American Dental Hygienists’ Association	1 member
National Association of Dental Laboratories	1 member
General Public	4 members
Students	1 member

(Reproduced from http://www.ada.org/en/coda/accreditation/coda-membership/).

## Conclusions

As the primary form of accreditation of dental education, dental education evaluation in China is a new evaluation system, it is basically suitable for current dental education conditions in China, and will be improved in due course as it progresses on.
